# Hepatic Fascioliasis Complicated by Multisite Venous Thromboembolism: A Rare Association and Implications for Parasite‐Associated Coagulopathy

**DOI:** 10.1002/ccr3.70647

**Published:** 2025-07-21

**Authors:** Girma Deshimo Lema, Seife Feleke Mulatu, Zena Admasu Yferu, Getachew Bizuneh Aydagnuhm, Wogderes Bogale Gebresillassie, Yidersal Demsie Denberu, Asrat Berihun Dagnaw, Ermias Fikru Yesuf, Enguday Demeke Gebeyaw

**Affiliations:** ^1^ School of Medicine, Asrat Woldeyes Health Science Campus Debre Berhan University Debre Berhan Ethiopia; ^2^ School of Public Health, Asrat Woldeyes Health Science Campus Debre Berhan University Debre Berhan Ethiopia

**Keywords:** coagulopathy, eosinophilia, *Fasciola hepatica*, multisite venous thromboembolism, parasite‐associated thrombosis

## Abstract

Hepatic fascioliasis is a trematode flatworm infection caused by *Fasciola hepatica* or *Fasciola gigantica* . Its association with venous thrombosis is exceedingly rare and can lead to significant morbidity when complicated by such conditions. Here, we report a rare case of hepatic fascioliasis complicated by multisite venous thromboembolism (VTE), highlighting the potential link between parasitic infections and coagulopathy. A 41‐year‐old woman presented with right leg swelling of 3 days duration. Examination revealed hypoxemia, tachypnea, and unilateral leg swelling. Laboratory tests showed marked eosinophilia, while imaging revealed multiple hypovascular focal hepatic lesions suggestive of *Fasciola hepatica* , alongside acute thrombosis of the right branch of the portal vein, iliac and femoral veins, and bilateral pulmonary emboli. Stool examinations were negative, but *Fasciola hepatica* IgG serology was elevated. The patient later developed right upper quadrant pain and a drop in hematocrit; repeat imaging showed sub‐capsular and parenchymal hepatic hematomas, likely related to parasite‐induced liver damage and anticoagulation. She was managed with antiparasitic therapy, anticoagulation, blood transfusion, and supportive care, with good recovery. This case underscores the need to consider fascioliasis in patients from endemic regions presenting with unexplained eosinophilia and venous thrombosis. It illustrates a rare but serious complication and highlights the importance of early diagnosis, integrated management, and further research into parasite‐associated coagulopathy.

**Abbreviations**
CT: computed tomography
VTE: venous thromboembolism

**Summary**

- This case emphasizes the importance of considering *Fasciola hepatica* infection as a potential cause of unexplained eosinophilia and venous thrombosis, particularly in endemic areas.
- While hepatic fascioliasis typically presents with hepatobiliary symptoms, it can present atypically with thromboembolism.
- Enhanced awareness, early diagnosis and integrated management are crucial in such cases.

## 1 Introduction

Hepatic fascioliasis is a trematode parasitic infection caused by *Fasciola hepatica* or *Fasciola gigantica* , commonly found in regions where livestock graze on contaminated water plants [1, 2]. Infection is prevalent in various regions, including Central and South America, Europe (notably Portugal, France, Spain, and Turkey), Asia, as well as Africa and the Middle East [3, 4]. It is estimated that between 2.4 and 17 million people are infected across more than 51 countries, with 180 million individuals at risk globally [5, 6]. The disease harms livestock, impacting farmers and industry with significant economic loss [3, 7]. It is also an emerging or re‐emerging parasitic disease with a significant global impact on human health [8, 9, 10].

Sheep and cattle are the main hosts of *Fasciola hepatica* , though other animals like goats, buffalo, and deer can also be infected. Snails serve as intermediate hosts, while humans are accidental hosts. Human infection follows consumption of plants or drinking water contaminated with infective metacercarial cysts of *Fasciola* [5, 9, 11, 12]. Transmission may also occur via ingestion of water containing free‐floating metacercariae or through contaminated vehicles such as soups, smoothies, or traditional emollient drinks [13, 14]. The parasite primarily affects the liver, causing inflammation, biliary obstruction, and fibrosis, with clinical manifestations ranging from mild abdominal discomfort to severe hepatobiliary disease [10, 12, 15, 16]. While liver fibrosis associated with *Fasciola hepatica* has been well documented in livestock, its occurrence in human cases remains to be fully established, though research suggests a possible link [10, 17].

While hepatic fascioliasis is a well‐documented cause of liver pathology, systemic complications, including venous thrombosis, are rare and underrecognized. The association between parasitic infections and coagulopathy has been an area of growing interest, with *Fasciola hepatica* being implicated in thrombotic events due to its ability to alter coagulation pathways [18, 19]. The risk of venous thromboembolism in patients with hepatic fascioliasis, however, remains poorly understood, and the potential for multisite thrombosis complicating the clinical course is an even rarer manifestation.

This case report presents a patient with hepatic fascioliasis complicated by multisite venous thromboembolism, offering insights into the clinical challenges of diagnosing and managing this rare and complex association. By exploring the potential mechanisms of parasite‐associated coagulopathy, this case underscores the importance of considering parasitic infections as a contributing factor in thrombotic events and emphasizes the need for further investigation into the pathophysiology of such rare associations.

## 2 Case History/Examination

A 41‐year‐old female patient presented to Debre Berhan University Hakim Gizaw Hospital in Northeast Ethiopia with a 3‐day history of right leg swelling. She is a known asthmatic with controlled symptoms, taking salbutamol puff and beclomethasone puff as needed. She denied experiencing cough, chest pain, bowel habit change, or any recent trauma. She has no history of smoking, and her family history was unremarkable. Notably, she reported a history of consuming raw aquatic vegetables.

During the physical examination, the patient presented with a respiratory rate of 28 breaths per minute and an oxygen saturation of 88% on room air. Chest auscultation revealed scattered bilateral wheezing in the posterior lung fields. Musculoskeletal assessment showed significant swelling of the right leg, with a 5 cm increase in circumference below the tibial tuberosity compared to the left leg. All other examinations were normal.

## 3 Differential Diagnosis, Investigations, and Treatment

Initial laboratory investigations showed a white blood cell count of 8290 cells per microliter, with an elevated absolute eosinophil count of 2487 cells per microliter. Hematocrit and platelet count were within the normal range. The erythrocyte sedimentation rate (ESR) was elevated at 61 mm/h. Liver function tests, including bilirubin and other chemistry parameters, were within normal limits, except for a mild elevation in alkaline phosphatase (136 IU/L; reference range: 40–50 IU/L). However, gamma‐glutamyl transferase (GGT) testing was not available in our setting. Blood glucose and other biochemical parameters were also normal. Stool analysis, performed twice during the initial admission using conventional microscopy and occult blood testing, showed no significant findings. Tests for human immunodeficiency virus (HIV), hepatitis B, and hepatitis C were negative. Serological tests for Venereal Disease Research Laboratory (VDRL) and antinuclear antibody (ANA) also returned negative results.

Doppler ultrasound of the lower extremity revealed an acute proximal deep vein thrombosis involving the right external iliac, femoral, and popliteal veins. Abdomino‐pelvic ultrasound showed a 3 cm × 3 cm heterogeneously hypoechoic focal lesion in the right lobe of the liver, along with acute thrombosis of the right portal vein branch. The chest X‐ray finding was unremarkable.

Given the initial clinical presentation and imaging results, anticoagulation therapy was initiated with warfarin 5 mg orally once daily and unfractionated heparin 12,500 IU subcutaneously twice daily, accompanied by oxygen support and ongoing management of bronchial asthma. The nature of the liver lesion remained inconclusive at this stage.

**FIGURE 1:**
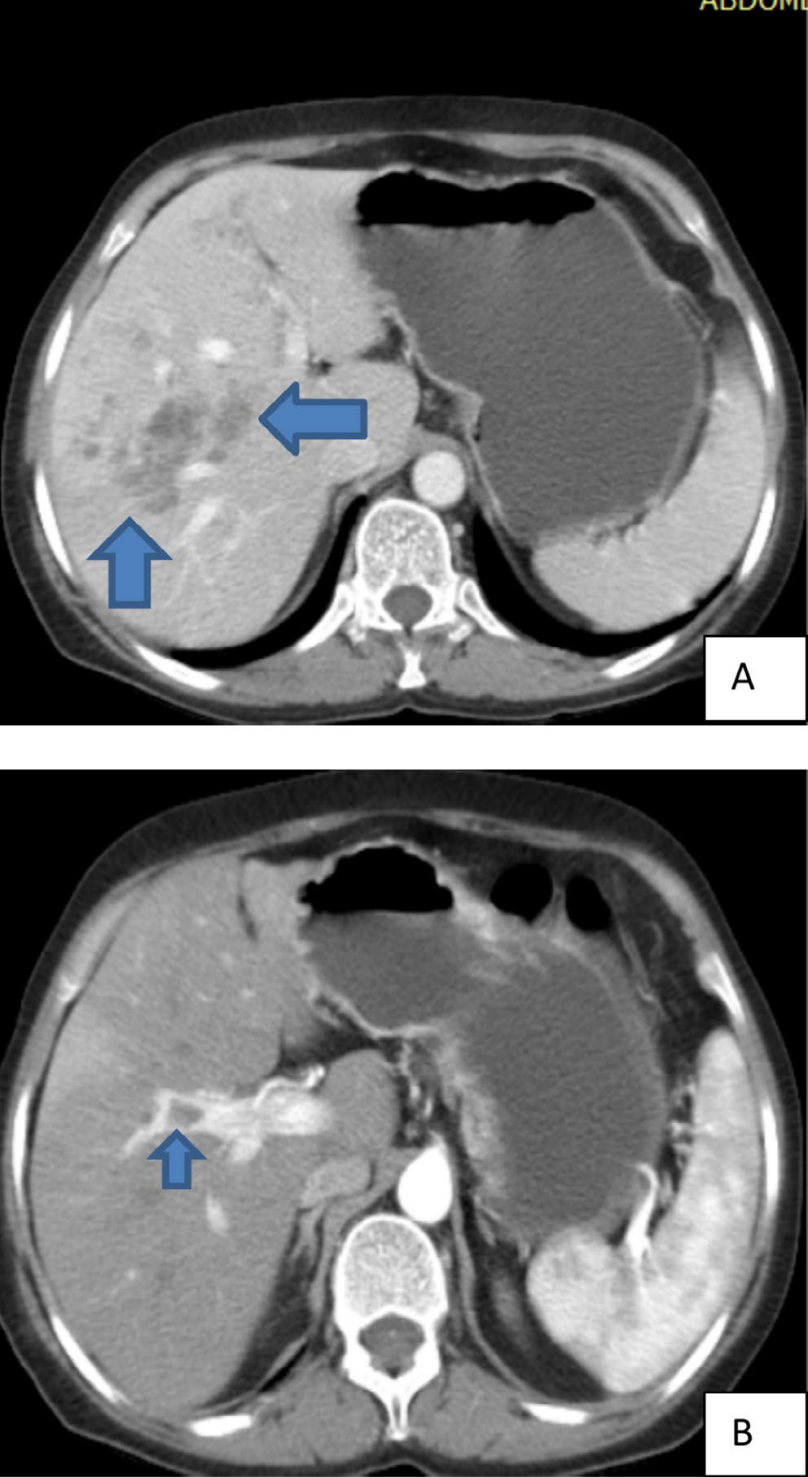
(A, B) Contrast‐enhanced abdominal computed tomography: (A) multiple hypo‐enhancing focal hepatic lesions (arrows) noted within the liver parenchyma, most appearing along the portal tracts; (B) Focal filling defect noted within the right anterior segment of portal vein (arrow).

**FIGURE 2:**
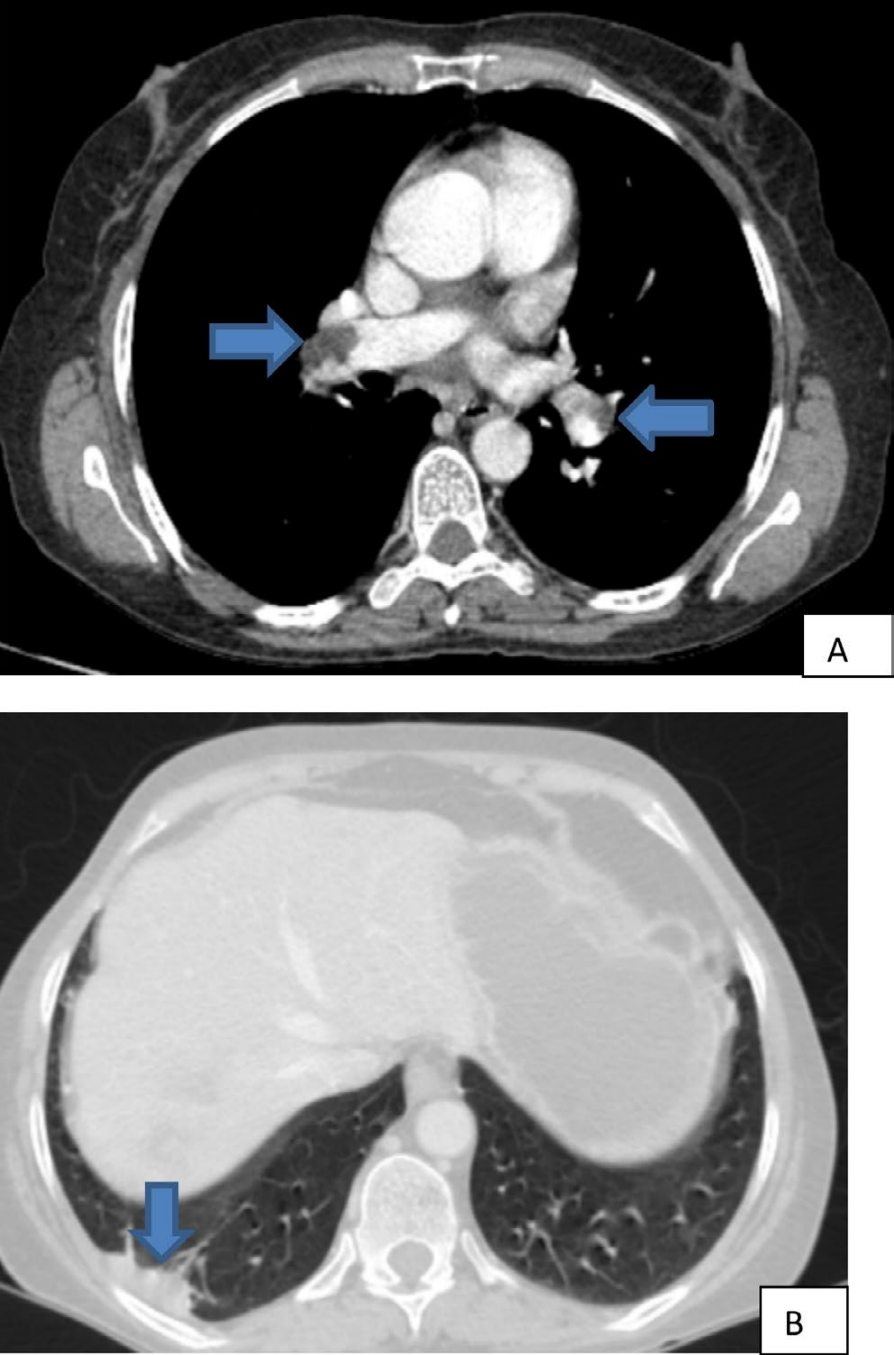
(A, B) Contrast‐enhanced chest computed tomography: (A) filling defect noted within the right main pulmonary artery and within the left descending pulmonary artery; (B) peripheral based air space opacity noted in the right basal segment of the lower lobe.

**FIGURE 3:**
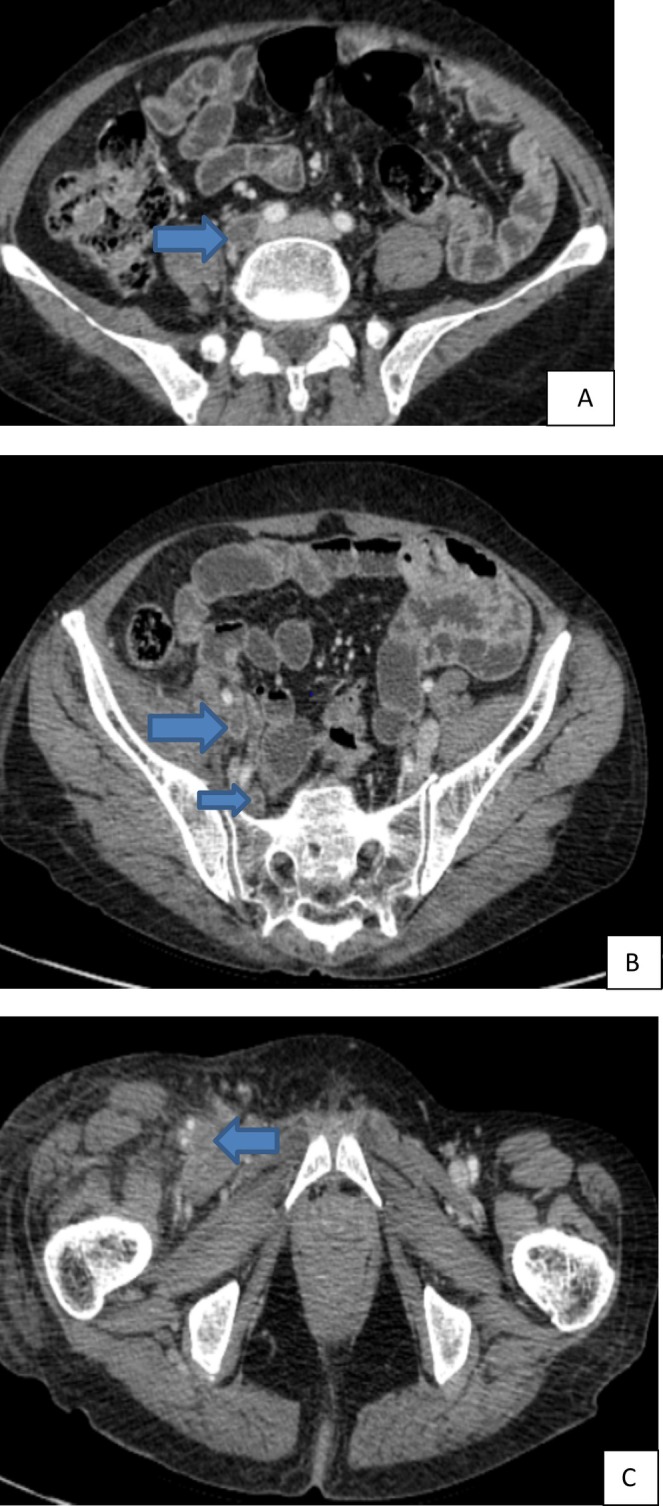
(A–C) Abdomino‐pelvic computed tomography showed: (A) a filling defect through the right common iliac vein; (B) internal (small arrow) and external (large arrow) iliac veins; and (C) right femoral vein.

On the second day of admission, contrast‐enhanced abdomino‐pelvic and chest computed tomography (CT) scans were performed at a nearby private center to investigate potential underlying causes such as malignancy and to further characterize the liver lesion. The imaging revealed multiple hypovascular focal hepatic lesions clustered along the portal tracts, findings highly suggestive of *Fasciola hepatica* infection (Figure 1A). Additionally, there was evidence of an acute thrombus in the anterior segment of the right portal vein (Figure 1B), bilateral acute pulmonary emboli with infarction in the right lower lobe (Figure 2A,B), and acute venous thrombosis involving the right common iliac vein, internal and external iliac veins (Figure 3A,B), and the right femoral vein (Figure 3C). The *Fasciola hepatica* IgG serology was elevated (17.7 U/mL, reference range < 9.0 U/mL), and a diagnosis of *Fasciola hepatica* complicated by multisite VTE was made. Albendazole 400 mg orally twice daily for 7 days was administered, as triclabendazole was not initially available.

On the fifth day of her admission, the patient complained of severe right upper abdominal pain and dizziness. Her hematocrit dropped significantly from 37.6% to 20.4%, and her abdominal ultrasound showed a significant increase in the liver lesion. The white blood cell count also increased to 11,100 cells per microliter, along with an elevated absolute eosinophil count of 3996 cells per microliter. The International Normalized Ratio (INR) was 3, which is within the therapeutic range. Complicated *Fasciola hepatica* infection with bleeding and hematoma was considered. Cross‐matched blood was transfused, and anticoagulation was withheld. As the patient's condition became critical, she was referred to a tertiary care center for better management and further investigations.

At the referral center, a repeat CT scan was performed, showing Fasciola infection complicated by a sub‐capsular hematoma (16 × 5.4 cm) and a parenchymal hematoma (6 × 3.5 cm), located in the right hepatic lobe. We were unable to obtain the actual CT images from the referral center. Our interpretation of the hematoma location is based on the radiologist's report rather than direct image review. Triclabendazole was administered at a dose of 10 mg/kg, given in two doses 12 h apart, and the patient was managed conservatively for the hematoma. After clinical stabilization and no further signs of bleeding, as evidenced by a rise in hematocrit and a decrease in eosinophil count, warfarin 5 mg orally once daily was reinitiated.

Further workup for thrombophilia and malignancy was conducted approximately four weeks after the initial presentation to help narrow potential confounders. Tumor markers, including CA 19–9, CEA (serum), Alpha‐Fetoprotein (AFP), as well as autoimmune and thrombophilia‐related tests such as anti‐beta‐2 glycoprotein I, anticardiolipin antibodies, and quantitative ANA, were all within normal limits and unremarkable.

## 4 Outcome and Follow‐Up

She was informed about her condition, showed clinical improvement, and was discharged with an outpatient follow‐up appointment and a prescription for warfarin 5 mg to be taken orally once daily. During her outpatient follow‐up visit, serial eosinophil counts and abdominal ultrasound showed progressive improvement. At four and eight weeks after the initial presentation, her absolute eosinophil count had decreased to 551 and 486 cells per microliter, respectively. A stool examination performed in the fifth week after the initial presentation was unremarkable. Follow‐up imaging was limited to serial ultrasound, which showed progressive decrement of the hepatic lesion, with the most recent scan performed at the fourth month after the initial presentation.

## 5 Discussion

The global distribution of *Fasciola hepatica* makes it a significant public health concern, particularly in areas where humans and livestock share water sources contaminated by the parasite's larvae [3, 9, 10, 20].

Fascioliasis is often mild or asymptomatic, with about half of the cases showing no symptoms. Disease severity increases with parasite load and progresses through two phases: acute (liver) and chronic (biliary). The acute phase typically presents with fever, right upper quadrant pain, hepatomegaly, and sometimes jaundice or urticaria. Marked peripheral eosinophilia is almost always present. The acute phase can be complicated by hemobilia or sub‐capsular hematomas of the liver [21, 22, 23, 24]. The chronic phase may be silent or involve abdominal pain, jaundice, and digestive issues, with possible complications like bile duct obstruction, cholangitis, gallstones, pancreatitis, or biliary cirrhosis. Eosinophilia is less common in chronic cases [17, 25, 26].

The patient in this case presented with significant venous thrombosis involving the right portal vein branch, iliac veins, femoral veins, popliteal veins, and pulmonary embolism. To the best of our knowledge, there are no published case reports in the literature documenting venous thromboembolism specifically associated with *Fasciola hepatica* infection. This case report highlights a rare complication of hepatic fascioliasis, multisite VTE, suggesting a potential connection between this parasitic infection and coagulopathy. The association between *Fasciola hepatica* and thrombosis is not well‐established in the literature, but there are emerging reports that indicate parasitic infections can lead to hypercoagulability. The exact mechanism by which *Fasciola hepatica* contributes to thrombosis remains unclear, but several factors may be involved. One potential mechanism is the secretion of cathepsin L proteinases by the parasite [18], which may induce inflammation and alter coagulation pathways, leading to the development of thrombosis. The increased eosinophil count observed in this case may further contribute to a prothrombotic state. Eosinophils have been implicated in modulating immune responses, and their activation can potentially impact the coagulation system by promoting thrombus formation and enhancing vascular endothelial injury [19].

The patient's condition was further complicated by severe abdominal pain and a drop in hematocrit, suggestive of possible bleeding secondary to liver involvement, potentially aggravated by anticoagulation therapy. Given the timing, imaging findings, clinical course, and the absence of alternative bleeding sources, it is highly plausible that *Fasciola hepatica* played a central role in predisposing the liver to hematoma formation. Additionally, with an INR of 3, an anticoagulation‐induced hematoma is also highly likely. *Fasciola hepatica* in its hepatic phase migrates through the liver parenchyma, causing direct tissue damage, necrosis, and inflammation. This invasive phase can lead to sub‐capsular hematomas and liver parenchymal hemorrhage [21, 27, 28, 29, 30].

Fascioliasis can pose a diagnostic challenge due to its varied and often nonspecific presentation. There is no single gold standard for its diagnosis; rather, it requires a multifaceted approach that combines clinical evaluation, laboratory testing, and imaging studies [3, 31, 32]. In this case, the initial presentation was atypical, with the patient experiencing unilateral leg swelling (a diagnosis of deep vein thrombosis). As part of the deep vein thrombosis workup, abdominal imaging was performed to evaluate for possible underlying causes, including occult malignancy or compressive pathology. Unexpectedly, this led to the detection of hepatic lesions, prompting further evaluation with contrast‐enhanced CT, which revealed multiple, hypovascular focal hepatic lesions, clustered along the portal tracts, a radiological hallmark suggestive of hepatic fascioliasis [33]. The initial presentation was atypical in our case, though she developed right upper quadrant abdominal pain 5 days after the initial presentation.

Although the patient had no typical hepatobiliary symptoms at presentation, she developed severe right upper quadrant abdominal pain 5 days later. A repeat contrast‐enhanced CT at that time demonstrated disease progression, now complicated by sub‐capsular and parenchymal hematomas, consistent with the acute hepatic migratory phase of *Fasciola hepatica* infection. Stool examinations were repeatedly negative, which is not uncommon, particularly in the early phase of infection before egg shedding begins.

Given the rarity of multisite VTE in fascioliasis, a comprehensive workup was conducted at the referral center in addition to the initial investigation to exclude other causes of thrombosis. Malignancy, autoimmune disease, and inherited or acquired thrombophilias were all ruled out based on normal tumor markers (CA 19–9, CEA, AFP), ANA, antiphospholipid antibody panel, and imaging findings.

Ultimately, the diagnosis was guided by highly suggestive imaging findings and peripheral eosinophilia, with serologic testing providing supportive evidence. This underscores the diagnostic value of imaging and eosinophilia, complemented by serology, in cases of suspected fascioliasis when stool microscopy is non‐diagnostic.

Stool microscopy has limited sensitivity, particularly during the early hepatic phase or in chronic infections with low parasite burden, as egg shedding typically begins only after the parasites mature in the biliary system. In our case, stool examination was performed three times during admission and once during follow‐up using conventional ova and parasite microscopy. More sensitive techniques, such as Kato‐Katz or other methods, were not available in our setting, which may account for the absence of detectable eggs.

The treatment of fascioliasis primarily involves anthelmintic therapy, with the specific approach tailored to the clinical presentation. Triclabendazole is the drug of choice due to its effectiveness against both adult and immature stages of the parasite. In cases where triclabendazole is unavailable or contraindicated, bithionol and nitazoxanide may serve as alternative options [34, 35]. Biliary decompression may be considered when clinically indicated, particularly in the presence of obstructive complications [16, 36, 37, 38]. Our patient's management included antiparasitic therapy, anticoagulation therapy for venous thromboembolism, and conservative treatment for hepatic hematoma, which involved blood transfusion, pain control, and bed rest.

## 6 Conclusion

This case highlights a rare but clinically significant complication of hepatic fascioliasis, multisite venous thromboembolism, pointing to a potential link between parasitic infections and hypercoagulable states. Although fascioliasis typically presents with hepatobiliary symptoms, its initial manifestation as thrombosis in this patient underscores the need for high clinical suspicion, especially in endemic areas or in the presence of unexplained eosinophilia. The development of hepatic hematomas, likely worsened by anticoagulation, illustrates the possible risk of severe complications during the acute hepatic phase. Timely administration of triclabendazole and individualized management are crucial for a favorable outcome. This case adds to the limited literature on Fasciola‐associated coagulopathy and highlights the need for further research into its pathophysiology and the management of its complications.

## Author Contributions

**Girma Deshimo Lema:** conceptualization, data curation, formal analysis, investigation, methodology, project administration, resources, supervision, validation, writing – original draft, writing – review and editing. **Seife Feleke Mulatu:** conceptualization, data curation, investigation, software, supervision. **Zena Admasu Yferu:** conceptualization, formal analysis, investigation, validation. **Getachew Bizuneh Aydagnuhm:** conceptualization, formal analysis, investigation, validation. **Wogderes Bogale Gebresillassie:** conceptualization, formal analysis, investigation, validation. **Yidersal Demsie Denberu:** data curation, methodology, supervision, validation. **Asrat Berihun Dagnaw:** data curation, methodology, supervision, validation. **Ermias Fikru Yesuf:** data curation, methodology, supervision, validation. **Enguday Demeke Gebeyaw:** formal analysis, investigation, methodology, writing – review and editing.

## Ethics Statement

Written informed consent was obtained from the patient for publication of this case report and any accompanying images.

## Conflicts of Interest

The authors declare no conflicts of interest.

## Data Availability

The data that support this case report are available in the manuscript of this article.
